# Fast 2D NMR Spectroscopy for *In vivo* Monitoring of Bacterial Metabolism in Complex Mixtures

**DOI:** 10.3389/fmicb.2017.01306

**Published:** 2017-07-14

**Authors:** Rupashree Dass, Katarzyna Grudzia̧ż, Takao Ishikawa, Michał Nowakowski, Renata Dȩbowska, Krzysztof Kazimierczuk

**Affiliations:** ^1^Centre of New Technologies, University of Warsaw Warsaw, Poland; ^2^Faculty of Chemistry, Biological and Chemical Research Centre, University of Warsaw Warsaw, Poland; ^3^Department of Molecular Biology, Faculty of Biology, Institute of Biochemistry, University of Warsaw Warsaw, Poland; ^4^Dr Irena Eris Cosmetic Laboratories, Centre for Science and Research Warsaw, Poland

**Keywords:** NMR spectroscopy, *Propionibacterium acnes*, non-uniform sampling, *in vivo* process monitoring, antimicrobial agent

## Abstract

The biological toolbox is full of techniques developed originally for analytical chemistry. Among them, spectroscopic experiments are very important source of atomic-level structural information. Nuclear magnetic resonance (NMR) spectroscopy, although very advanced in chemical and biophysical applications, has been used in microbiology only in a limited manner. So far, mostly one-dimensional ^1^H experiments have been reported in studies of bacterial metabolism monitored *in situ*. However, low spectral resolution and limited information on molecular topology limits the usability of these methods. These problems are particularly evident in the case of complex mixtures, where spectral peaks originating from many compounds overlap and make the interpretation of changes in a spectrum difficult or even impossible. Often a suite of two-dimensional (2D) NMR experiments is used to improve resolution and extract structural information from internuclear correlations. However, for dynamically changing sample, like bacterial culture, the time-consuming sampling of so-called indirect time dimensions in 2D experiments is inefficient. Here, we propose the technique known from analytical chemistry and structural biology of proteins, i.e., time-resolved non-uniform sampling. The method allows application of 2D (and multi-D) experiments in the case of quickly varying samples. The indirect dimension here is sparsely sampled resulting in significant reduction of experimental time. Compared to conventional approach based on a series of 1D measurements, this method provides extraordinary resolution and is a real-time approach to process monitoring. In this study, we demonstrate the usability of the method on a sample of *Escherichia coli* culture affected by ampicillin and on a sample of *Propionibacterium acnes*, an acne causing bacterium, mixed with a dose of face tonic, which is a complicated, multi-component mixture providing complex NMR spectrum. Through our experiments we determine the exact concentration and time at which the anti-bacterial agents affect the bacterial metabolism. We show, that it is worth to extend the NMR toolbox for microbiology by including techniques of 2D z-TOCSY, for total “fingerprinting” of a sample and 2D ^13^C-edited HSQC to monitor changes in concentration of metabolites in selected metabolic pathways.

## 1. Introduction

Many spectroscopic methods have been introduced into experimental practice of biological researchers in recent decades. Nuclear magnetic resonance (NMR) spectroscopy is unique, since it provides information about molecular structure at atomic level. In this form of spectroscopy a sample is placed in a strong homogeneous magnetic field. It is then excited using radio-frequency pulses which trigger the transitions between energy states of nuclear magnetic moments interacting with magnetic field. The excited nuclei relax back to equilibrium state and emit a decaying radio-frequency wave called the free induction decay (FID) signal. The Fourier transform of the FID produces a spectrum. Analysis of the peaks in this spectrum gives information about the sample such as the numbers and kinds of nuclei present in it, their chemical environments and mutual interactions through chemical bonds and space. This information can be used further to elucidate the chemical structure of the components present in the sample. Extending this further, NMR can also provide quantitative information on the relative concentrations of chemicals present in a complex chemical mixture. In addition, it can be used to monitor reactions to deduce information such as the mechanism and kinetics.

In biology, NMR is a long established technique to ascertain structures and conformations of proteins (Wüthrich, 2001), discern the function of numerous biomolecules (Forseth and Schroeder, 2012), screening and designing of drugs (Stockman and Dalvit, 2002), and in metabolomics research (Larive et al., 2015), among other applications. NMR has also been used successfully to investigate metabolism of microorganisms (Grivet et al., 2003; Halouska et al., 2013; Dzien et al., 2016). *In vivo* NMR is a rapidly emerging field in which new techniques are being constantly developed to study live cells (De Graaf, 2007).

**Figure 1:**
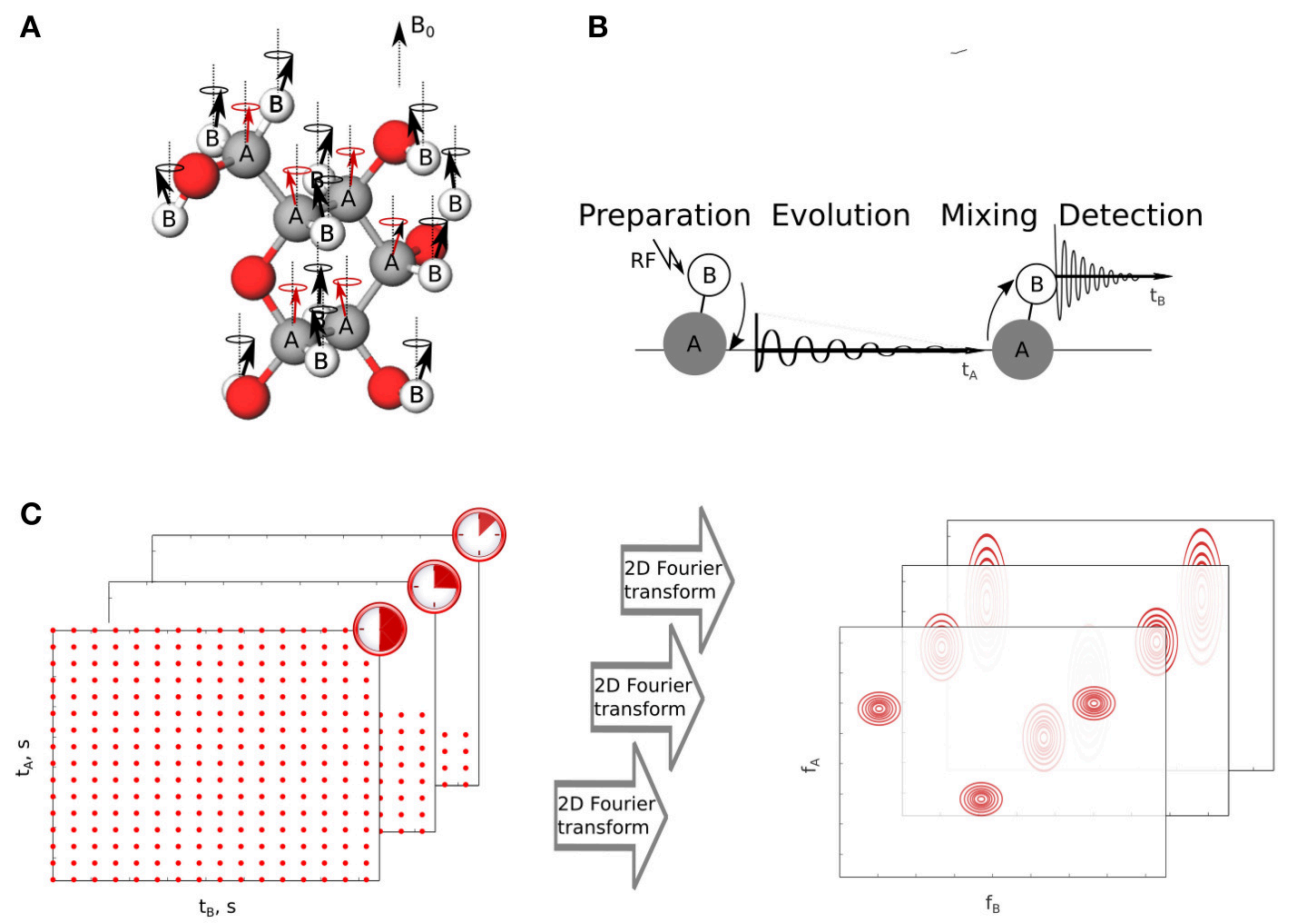
The idea of two-dimensional NMR experiment. **(A)** Nuclei of two elements A and B have non-zero spin and thus magnetic moments interact with external magnetic field *B*_0_. The interaction results in the incoherent precession of magnetic moments. **(B)** Excitation transfer between coupled nuclei A and B by two-dimensional NMR pulse sequence. **(C)** Sampling of a two-dimensional signal and its relation with spectral resolution. The longer evolution time sampled, the narrower spectral peaks in a sampled dimension. *t*_*A*_ and *t*_*B*_ stand for indirect and direct time domains, respectively. *f*_*A*_ and *f*_*B*_ are corresponding coordinates in frequency domain.

Most of NMR techniques have been established for stable samples, but in practice one often deals with uncontrolled, dynamic changes. Conventionally, to monitor them one acquires a series of simple one-dimensional (1D) ^1^H NMR spectra to evaluate the chemical reactions occurring during the process. For example, Blankenberg et al. (1996) and Blankenberg et al. (1997) have observed specific spectral changes that take place in a series of simple ^1^H spectra of bacterial suspension during the process of apoptic cell death. This has been further used to quantify cell death in both eukaryotic and prokaryotic bacteria (Hakumaki and Kauppinen, 2000; Cooper et al., 2001; Kettunen and Brindle, 2005; Schmitz et al., 2005; Hindmarsh et al., 2015). While ^1^H spectra are sensitive and quantitative in nature they do not provide enough spectral resolution to resolve signals from components of a complicated mixture. Multidimensional NMR experiments can circumvent this problem by resolving the peaks in multiple dimensions and simplifying the obtained spectrum. In these experiments correlations are established between nuclear spins and the resulting spectrum gives information on the chemical bonding or spatial vicinity between these nuclear spins as shown in Figures 1A,B.

This benefit comes at the cost of increased experimental time, as sampling indirect time dimensions of multidimensional FID is not as simple as sampling real time signals. Nuclear magnetic moments excited in the experiment indirectly, i.e., obtaining energy from other, directly excited in the experiment, precess with their specific frequencies. The precession is sampled by changing the length of certain delay between RF pulses in a pulse sequence used to excite system of nuclei. Each sampling point in the indirect dimension is thus a separate NMR experiment and costs few seconds of real time, leading to hours-long experiments. It is noteworthy, that the reasons for long data collection are purely mathematical, independent of the sensitivity of instruments used. Namely, the spectral resolution (inverse of linewidth) is proportional to maximum evolution time sampled, which is known as “Fourier uncertainty principle” (Szántay, 2008). Since the distance between sampling points is determined by the size of a band of sampled frequencies (known as Nyquist–Shannon theorem; Nyquist, 1928), the resolution can be improved only by extending the experimental time (see Figure 1C).

**Figure 2:**
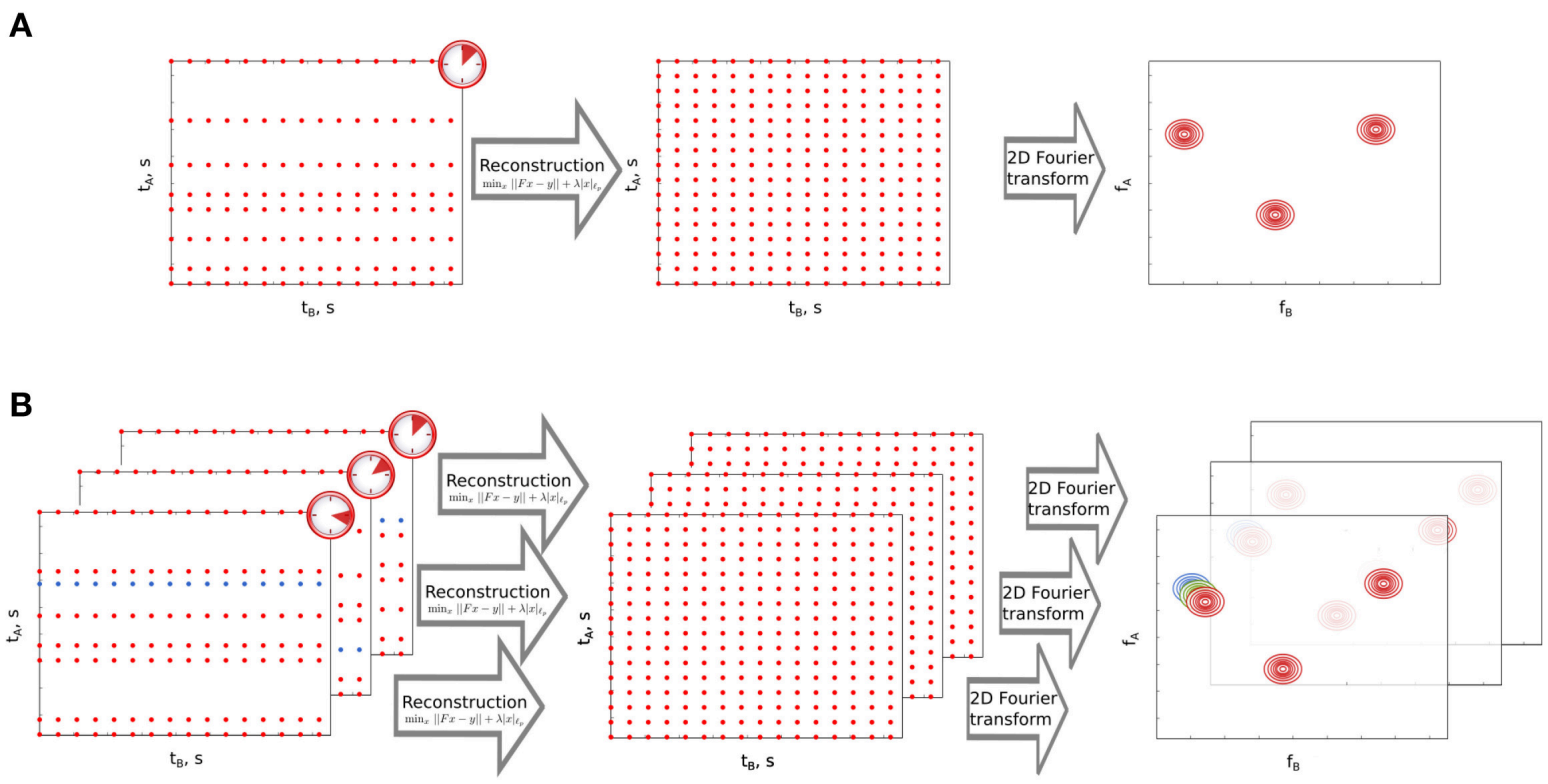
Non-uniform sampling (NUS) and its time-resolved variant. **(A)** Non-uniform sampling of the indirect time dimension in a 2D NMR experiment. *t*_*A*_ and *t*_*B*_ stand for indirect and direct time domains, respectively. *f*_*A*_ and *f*_*B*_ are corresponding coordinates in frequency domain. Omitted sampling points in *t*_*A*_ are reconstructed using sparsity-constrained algorithm. The experimental time is much shorter than in the case of full sampling. **(B)** Time-resolved NUS. Consecutive datasets are created by sampling one new point in *t*_*A*_ (marked in blue) and removing the oldest point from the preceding dataset. NUS datasets are processed as in A and form a stack of spectra. Peak marked with colors moves under the effect of reaction occurring in the sample.

Non-uniform sampling (NUS) is a technique to acquire multidimensional NMR experiments in much shorter time without compromising on resolution. In this technique one samples only a fraction of data points in the indirect dimensions and the missing data points are reconstructed using various algorithms, each differing from the other in its assumptions about the properties of the reconstructed spectrum. These assumptions may include maximum entropy (Mobli and Hoch, 2008), models of a spectrum (Mandelshtam, 2000; Orekhov and Jaravine, 2011), or maximum sparsity (Holland et al., 2011; Kazimierczuk and Orekhov, 2011). The latter assumption follows a mathematical framework referred to as compressed sensing (CS), which assumes that the NMR spectrum has a significant amount of empty regions (Candes et al., 2005). In other words, it assumes that in an NMR spectrum, the number of points corresponding to peaks is much smaller than the total number of points in the entire spectrum. According to the theory, the number of points one has to sample should be proportional to the number of important points (peaks) in the spectrum. As a result, CS finds the sparsest spectrum from relatively small amount of data (the smaller, the sparser the spectrum is). Figure 2A shows the concept of CS processing of the NUS data.

As NUS can help to acquire high resolution spectra in a short time it has been used to study fast reactions (Wu et al., 2014). Orekhov and coworkers have shown a variant of NUS i.e., time-resolved non-uniform sampling (TR-NUS) that can be used for monitoring fast processes in a continuous manner (Mayzel et al., 2014b). In this case sampling of the data points occurs in parallel to the reaction occurring in the NMR tube. The sampling schedule is shuffled and repetitive set of points which is long enough to cover the entire duration of the reaction. Subsets of data points are formed and each subset is processed using multidimensional decomposition (MDD), a high-dimensional variant of principal component analysis (Orekhov et al., 2017) to reconstruct a spectrum. We have further modified TR-NUS by introducing CS processing, that contrary to MDD, does not require stable peak positions (Bermel et al., 2014; Dass et al., 2015, 2016). The algorithm was used to reconstruct missing points in every overlapping subset and obtain a huge stack of two-dimensional (2D) spectra. In this stack, the reaction time acts as the third pseudo-dimension. This enables one to see in a “movie-like” manner various chemical changes that occurred in the sample. We have previously applied this method to monitor reactions occurring in natural mixtures (Dass et al., 2015) and the process of protein unfolding with rising temperature (Bermel et al., 2014). Figure 2B presents the concept of TR-NUS with CS processing.

In this paper, we show how two-dimensional NMR with TR-NUS can be used as a part of biological toolbox to monitor the bacterial cells in the presence of an antibacterial agent. As the first example we use *Escherichia coli* culture affected by ampicillin. As the second we show how *Propionibacterium acnes*, an acne-causing bacteria react to the commercially available cosmetic product that claims to inhibit their growth. We discuss the applicability of various kinds of 2D NMR experiments with TR-NUS to monitor the entire process in a continuous manner. The effect of the various ingredients of the cosmetic product on the bacterial metabolism has also been discussed. We believe that such *in vivo* process-monitoring techniques can help in understanding microbial metabolism and designing appropriate antimicrobial agents. Being as easy to set up as conventional series of 1D spectra, they provide more complete information about the phenomena occurring in the sample.

## 2. Materials and methods

### 2.1. Cultivation of *Escherichia coli* and *Propionibacterium acnes*

*E. coli* XL-1 Blue MRF' was cultivated overnight in LB medium (1% NaCl, 0.5% yeast extract, 1% tryptone, pH 7.5) at 37°C with shaking (250 rpm).

*P. acnes* ATCC 11827 was cultivated in TSB medium with 5.0% sheep blood (tryptic soy broth; 1.5% tryptone, 0.5% soytone, 0.5% NaCl, 5% sterile sheep blood, pH 7.3) in microaerophilic conditions (6.0% oxygen) at 37°C for 5 days. The volume of typical bacterial culture was 30 ml.

### 2.2. Preparation of NMR sample

To prepare one sample of *E. coli* for NMR experiments, 6 ml of culture in LB medium was used. Bacterial cells were collected by centrifugation at 16,000× g for 5 min. then pellet was washed twice by sterile water, and finally resuspended in 600 μl of 1:9 D_2_O:H_2_O.

To prepare one sample of *P. acnes* for NMR experiments, bacteria cultivated in 15 ml of TSB medium were used. First, bacterial culture was transferred to Eppendorf tubes and centrifuged at 16,000 × g for 10 s. Next, the supernatant was transferred to fresh Eppendorf tubes and centrifuged at 16,000 × g for 5 min. The bacterial pellet was washed twice by sterile water and then resuspended in 600 μl of D_2_O.

The number of viable cells in NMR sample was determined by preparing its serial dilutions in sterile water and plating 100 μl of them on LB (*E. coli*) or TSB medium (*P. acnes*) supplemented with 1.8% agar-agar. Plates were incubated overnight (*E. coli*) or for 5 days (*P. acnes*) at 37°C. By counting bacterial colonies, we determined that NMR sample (600 μl) contains the same amount of 9.0 × 10^7^ viable cells both in the case of *E. coli* and *P. acnes*.

### 2.3. NMR experimental setup

To trigger the metabolism in the *E. coli* sample the 0.36% (w/v) (20 mM) glucose was used (^13^C-labeled glucose for HSQC experiment). The entire process was monitored over several hours using 2D ^13^C-HSQC and 2D z-TOCSY with TR-NUS. Then, second series of experiments was carried out for samples containing 0.14 mM ampicillin, in addition to 20 mM glucose.

Initially to *P. acnes* sample 0.6 μl of 10% (w/v) glucose solution was added and the fermentation process (propionic acid production; Grinstead and Barefoot, 1992) was studied *in situ* inside an NMR magnet. The entire process was monitored over several hours using 2D ^13^C-HSQC and 2D z-TOCSY with TR-NUS. A series of experiments were conducted by adding varying amounts of face tonic (0.6, 1.2, 1.8, 2.4 μl) making the concentration of face tonic: 0.1, 0.2, 0.3, and 0.4%, respectively in the sample. The face tonic is a bacteriostatic face cleaning solution : Sebo-Almond-Claris containing 2% of mandelic acid from Dr. Irena Eris S.A., Poland.

In a second series of experiments on *P. acnes* the amount of mandelic acid solution (2% or 131.6 mM) was varied (0.0, 0.6, 1.2 μl) making its concentration : 0, 0.002, 0.004% (or 0, 131.6, 263.2 μM) in the sample. In a third series of experiments the effect of laniceric acid, lappa root extract and willow bark extract was studied by adding 3 μl of 2% (v/v) solution of each agent to the sample (results shown in SI).

To monitor the metabolism of bacteria in the presence of the conditions mentioned above TR-NUS was implemented by replacing the conventional sampling with a long NUS schedule in z-TOCSY pulse sequence (with WALTZ-16 decoupling sequence) from VnmrJ 4.2 software. The mixing time in the z-TOCSY pulse sequence was 80 ms. Spectral widths of 6,983 and 11,160 Hz were used in the indirect and direct dimension, respectively. Pulse width of 7.1 μs and interscan delay of 1 s was used. Two thousand forty-eight non-uniformly sampled points were taken from a grid size of 256 points with 4 scans per point. Each experiment was run for 6 h. The experiments were performed on an Agilent 700 MHz DDR2 NMR spectrometer with VnmrJ 4.2 software, equipped with HCN probe. All *E. coli* the experiments were conducted at 310 K, while all *P. acnes* experiments at 298 K. The final experiment made on each sample with bacterial cells sedimented at the bottom of NMR tube revealed, that products are present in the supernatant.

The processing of the data was done in a time-resolved manner (Section 2.4). Overlapping subsets (“frames”) of 128 (both *P. acnes*) and 64 (both *E. coli*) points were made from the dataset of 2,048 points. Each subset was individually processed using iteratively re-weighted least squares (IRLS) algorithm from CS module of mddnmr 2.5 software (Orekhov et al., 2017) to produce a spectrum. Default processing parameters were used (20 iterations and virtual echo option (Mayzel et al., 2014a). A stack of 1,791 spectra was obtained, each spectrum corresponding to an averaging of 11 min of reaction time.

**Figure 3:**
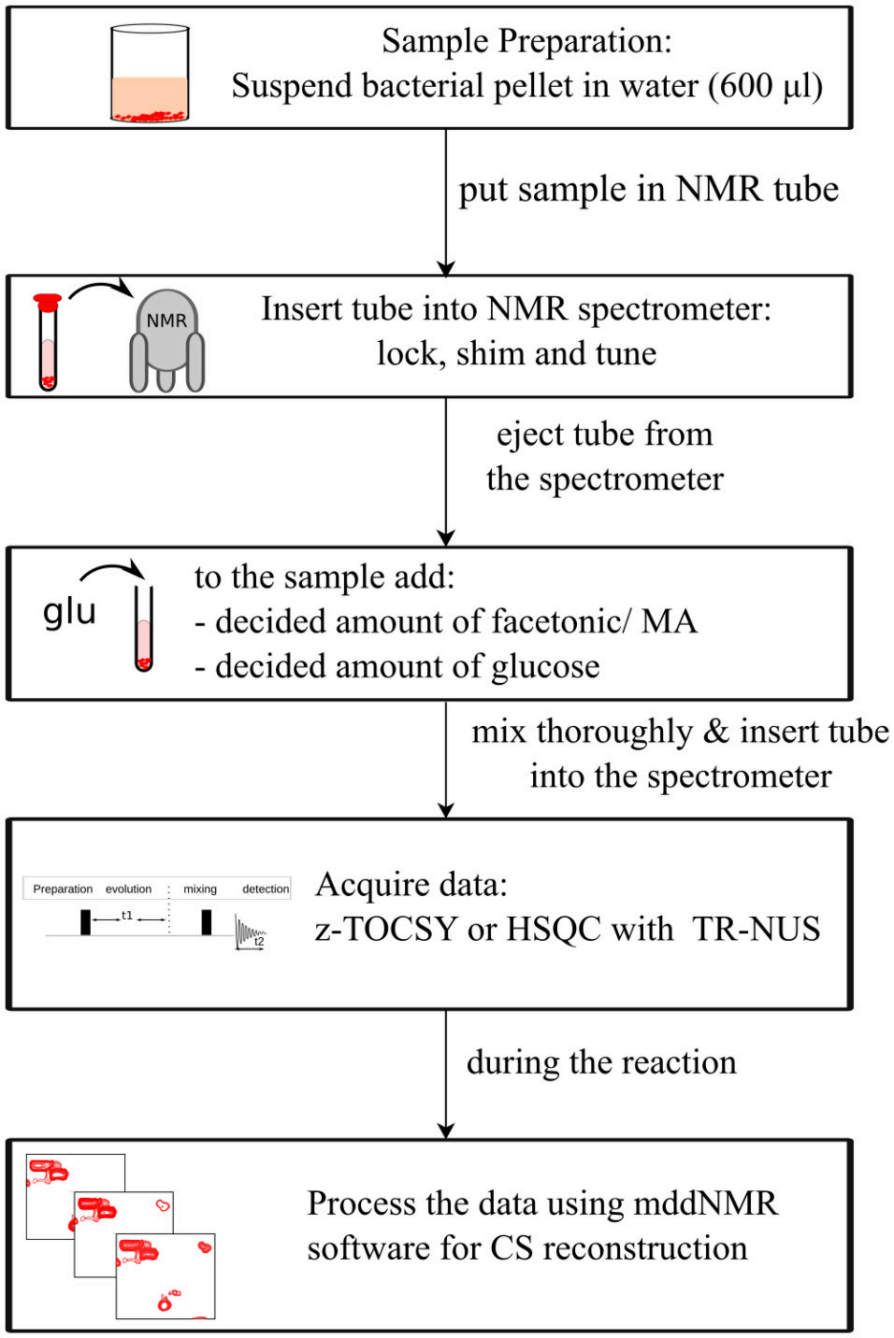
Scheme of the experimental setup.

To monitor products of glucose metabolism, uniformly enriched ^13^C-glucose was added to the solvent and the experiment was acquired for ca. 16 h. ^13^C-HSQC pulse sequence from VnmrJ 4.2 with WURST 140 adiabatic pulses sequence was used. Hard pulses of 7.1 μs (^1^H) and 12.1 μs (^13^C) were used. Spectral widths were kept equal to 12,019 Hz (^1^H) and 24,649 Hz (^13^C). Interscan delay of 1 s was used. Eight thousand one hundred and ninety-two NUS points were taken from a grid of 256 points with 4 scans per point. The processing parameters were the same as in case of z-TOCSY datasets above. A stack of 8,064 HSQC spectra was obtained spanning a duration of 16 h, each spectrum corresponding to an averaging of 9 min of reaction time. The scheme of experimental set-up is shown in Figure 3.

The number of biological replicates was limited to two: ^13^C HSQC and z-TOCSY serve as independent verifications of the process occurring in the same biological conditions.

### 2.4. Time-resolved non-uniform sampling

In TR-NUS, sampling of the signal is done in parallel to the process occurring in the sample. Due to this, it is important that the sampling schedule is long enough to cover the entire duration of the process. The sampling schedule is therefore a long array of repetitive and random points taken from the Nyquist grid. Overlapping subsets of data are formed and each individual subset is processed using CS reconstruction algorithm (Bermel et al., 2014). The size of the subset determines the temporal resolution. According to the CS theory, for optimal reconstruction the minimum number of sampling points in each subset should be proportional to the number of important peaks in the spectrum (Candes et al., 2005).

(1) n∝Klog(N/K)

Here, *n* is the number of points in each subset taken from a full grid of *N* points and *K* is the number of important points/peaks in the spectrum. Most importantly, one can optimize *n* after the acquisition of the data to balance between fulfilling CS condition (Equation 2) and avoiding too high averaging of spectral effects within a subset. Sometimes, especially in case of samples discussed in this work, low sensitivity (high signal-to-noise ratio) is limiting the sampling minimum more strictly than above condition. CS finds the sparsest spectrum that fits to the sampling by ℓ_*p*_-norm minimization :

(2) $$\underset{S}{\min}||iFT\cdot S-s||+\lambda |S{|}_{\ell_{p}}$$

Here, *S* is the spectrum, *s* is the non-uniformly sampled signal, *iFT* is the inverse Fourier transform matrix and λ is the parameter that keeps the balance between the consistency of the measured data and sparsity of the spectrum. This minimum can be determined e.g., by using the Iterative Re-weighted Least Squares (IRLS) algorithm (Lawson, 1961) from the mddnmr 2.5 software. The program *nussampler* from the mentioned package can be used to generate sampling schedules.

## 3. Results

**Figure 4:**
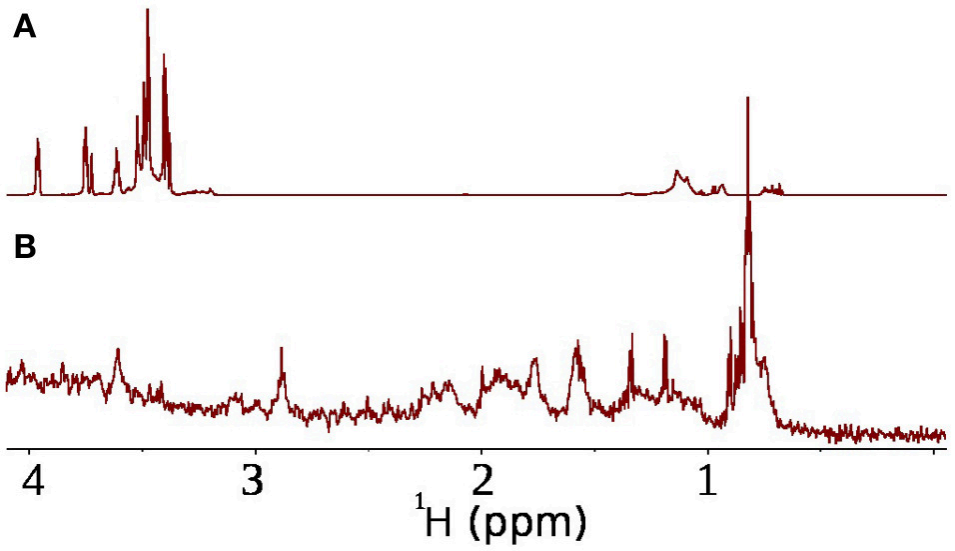
Potential problem with overlap of resonances from antimicrobial agent and bacteria. **(A)** ^1^H spectrum of face tonic (0.1%) in D_2_O (600 μl), **(B)** ^1^H spectrum of *P. acnes* bacteria in D_2_O.

The conventional approach to monitor bacterial activity *in situ* in a series of 1D experiments can be shown to be infeasible in a presence of multi-ingredient mixture, like face tonic. The ^1^H spectrum of face tonic and suspended bacterial pellet is depicted in Figures 4A,B. One can see the heavy overlapping of peaks originating from bacteria or their metabolites and peaks from the face tonic. To achieve increased resolution a series of 1D spectra can be replaced with 2D experiments.

**Figure 5:**
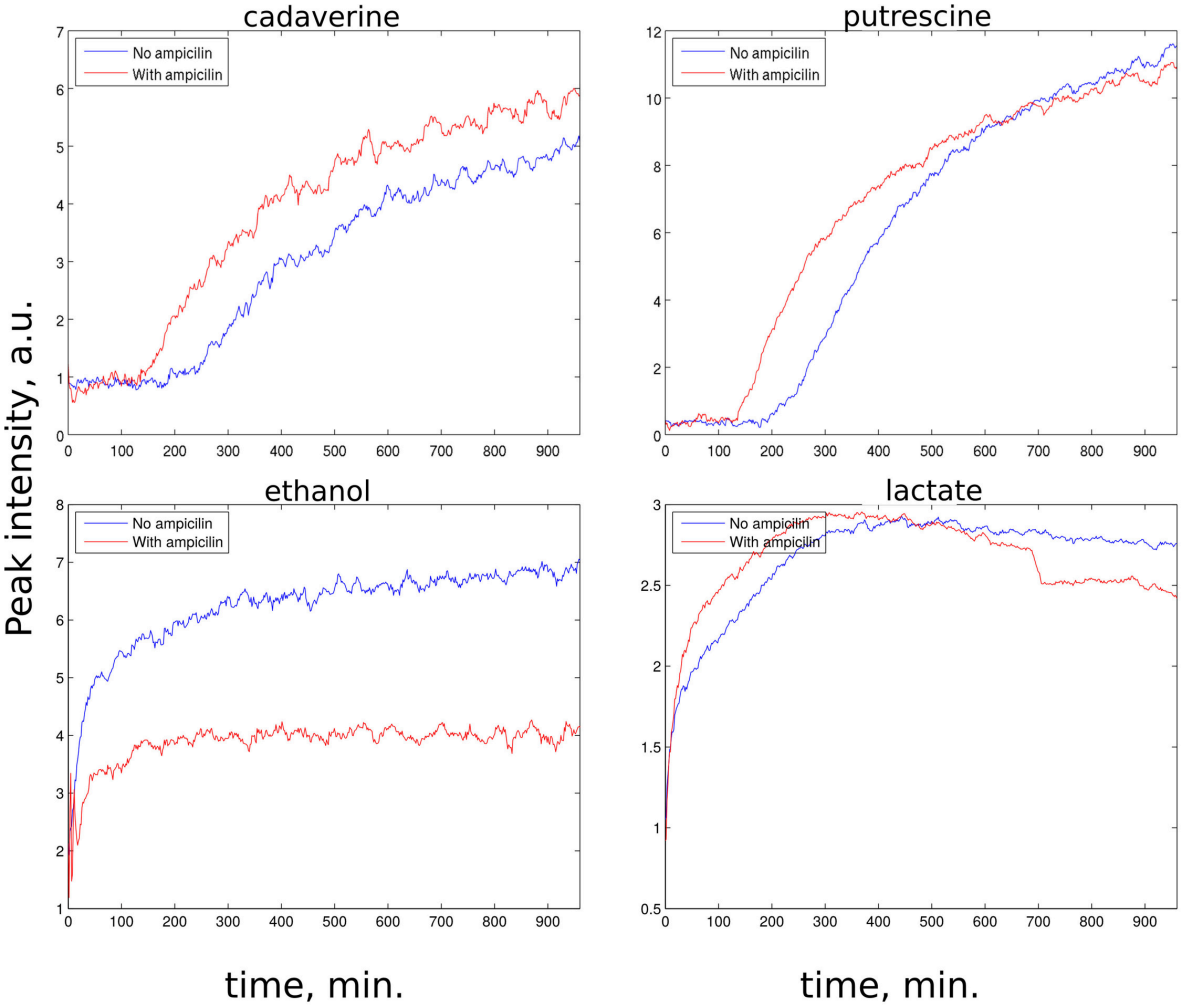
Rate of growth of products of *E. coli* metabolism in z-TOCSY spectra of samples with ampicillin (red) and without (blue). **(Upper left)**: growth of cadaverine peak at (1.71, 1.46 ppm), **(upper right)**: growth of putrescine peak at (3.06, 1.76 ppm), **(lower left)**: growth of ethanol peak at (1.17, 3.65 ppm), **(lower right)**: growth of lactate peak at (1.31, 4.1 ppm). Peaks are marked on the video shown in Supplementary Material.

**Figure 6:**
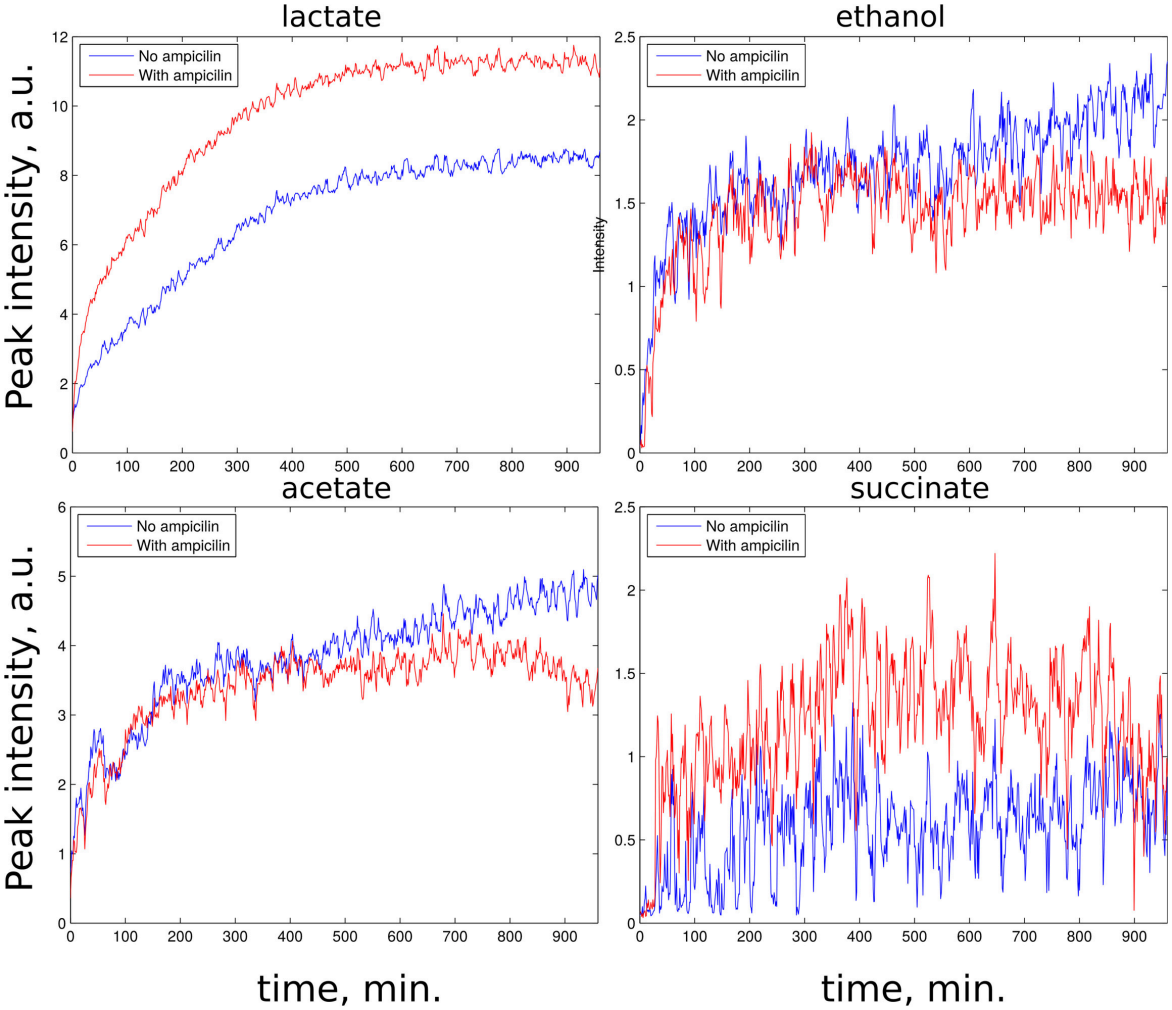
Rate of growth of products of *E. coli* metabolism in ^13^C HSQC spectra of samples with ampicillin (red) and without (blue). **(Upper left)**: lactate peak at (21.9, 1.30 ppm), **(upper right)**: ethanol peak at (18.7, 1.14 ppm), **(lower left)**: acetate peak at (23.9, 1.96 ppm), **(lower right)**: succinate peak at (33.2, 2.63 ppm). Peaks are marked on the video shown in Supplementary Material.

**Figure 7:**
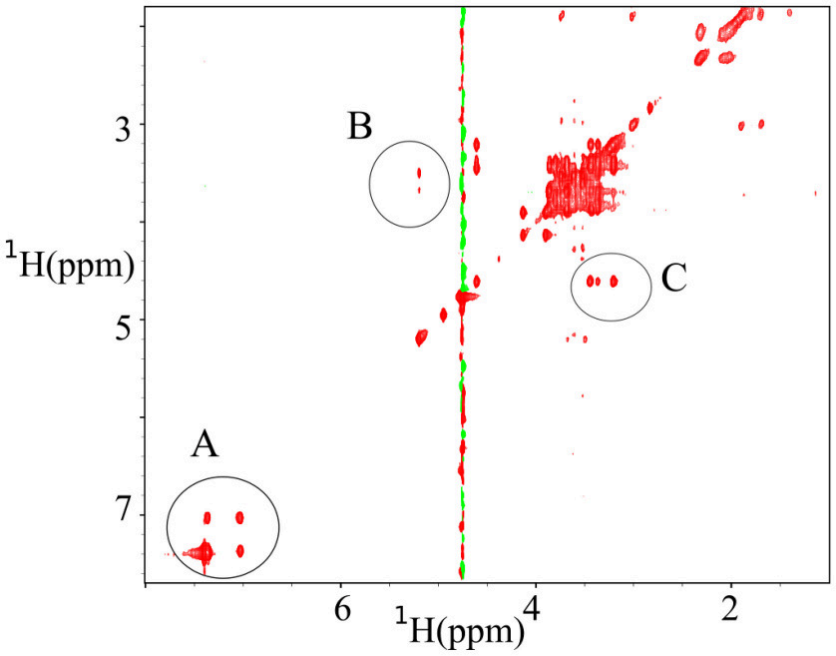
z-TOCSY spectra obtained 11 min after adding of 0.2% face tonic and 0.01% of glucose to a bacterial pellet of *P. acnes* in D_2_O. **(A)** Cross peaks from the aromatic protons of mandelic acid (active ingredient of the face tonic), **(B)** cross peaks from trehalose, and **(C)** Cross peaks from glucose.

**Figure 8:**
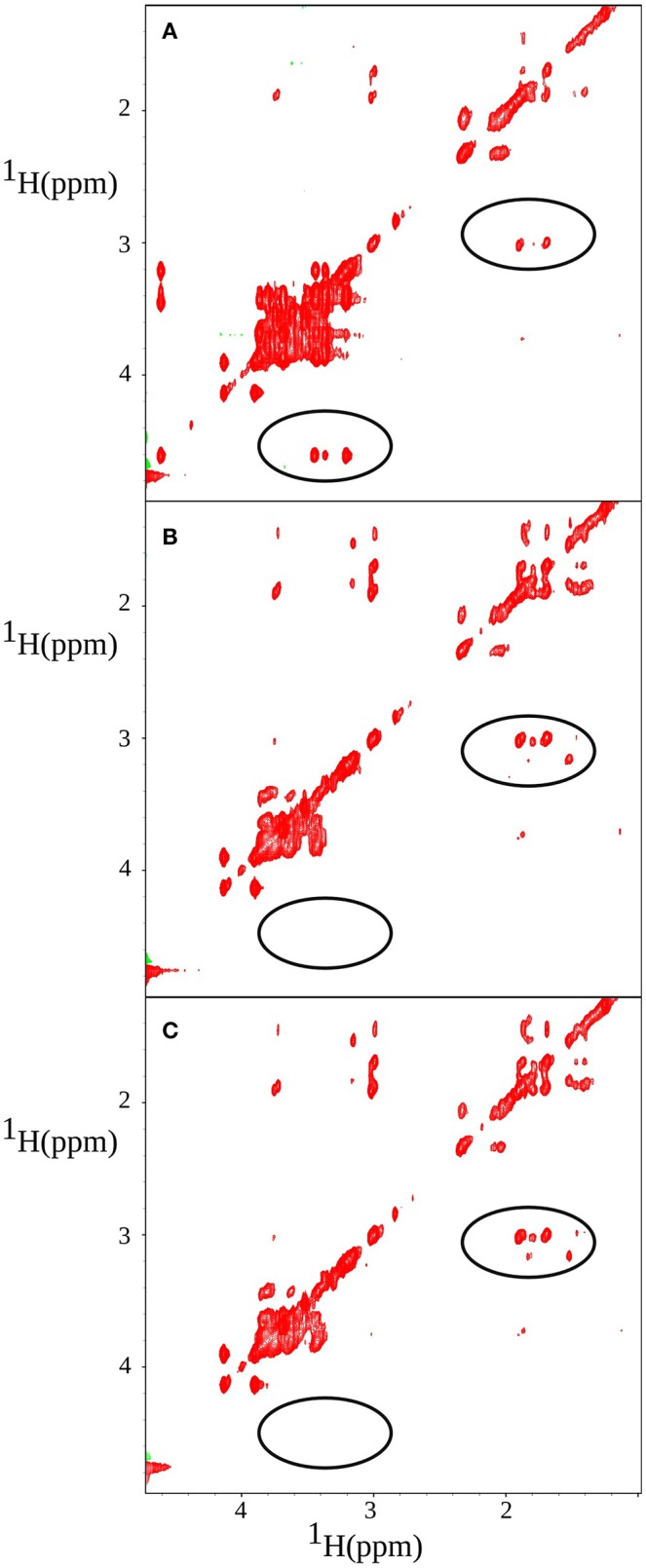
z-TOCSY spectrum obtained after adding of 0.2% face tonic and 0.01% of glucose to a bacterial pellet in D_2_O. Panels (from the top) correspond to **A** = 11 min, **B** = 139 min, and **C** = 349 min after the addition of face tonic. Regions of changes are marked with circles: glucose (left) and putrescine (right).

Before acquiring all experiments, the effect of D_2_O on the bacterial pellet of *P. acnes* was checked. z-TOCSY experiments containing only bacterial pellet suspended in D_2_O were measured (data not shown). No spectral changes were observed over time, showing that D_2_O does not affect bacterial cellular processes. In case of *E. coli* we used 10:90% D_2_O:H_2_O. These two different setups were used to draw attention to important practical aspect: the choice of 100% D_2_O provides spectra of better quality (no strong residual water signal), which is important for weak samples but some of the metabolites become deuterated and thus are invisible in z-TOCSY and ^13^C-HSQC spectra. The effect of D_2_O:H_2_O ratio on the deuteration of products of *E. coli* metabolism has been studied before by Griengl et al. (1999).

z-TOCSY spectrum of *E. coli* was acquired for samples with and without ampicillin. Figure 5 shows time plots of cadaverine, putrescine, ethanol, and lactate peak intensities, while video in Supplementary Material shows fragment of z-TOCSY spectrum with and without ampicillin. Intensities of marked peaks have been used for time plots. The products of cell decay, cadaverine and putrescine have been identified using TOCCATA web server (Bingol et al., 2014). Peaks from products of metabolism, e.g., ethanol and lactate can be also seen. However, not all products of *E. coli* metabolism give well-resolved off-diagonal peaks in z-TOCSY. For example, acetate or succinate give only diagonal peaks. However, they can be observed well in HSQC spectrum, as seen in videos in Supplementary Material and time plots in Figure 6.

z-TOCSY spectrum of *P. acnes* was acquired for a sample containing 0.2% face tonic, 0.01% of glucose (0.55 mM), and the bacterial pellet dissolved in D_2_O as shown in Figure 7. To be able to monitor bacterial activity with good temporal resolution with 2D experiments, we applied TR-NUS. The resulting stack of 2D “frames” shows changes in the spectra over time of the process of interaction with face tonic. Figure 8 (as well as a movie in SI) shows 2D frames of a small part of the spectrum at various time points obtained from the z-TOCSY experiments with TR-NUS. All peaks are well resolved showing the metabolomic changes occurring in the sample with time. The high signal-to-noise ratio shows that the 128 points frame size kept during NUS processing is optimal (for the discussion of the frame size see Dass et al., 2015). The sample in this case contains 0.01% (0.55 mM) glucose and 0.1% face tonic. One can observe the strong glucose peaks in the chemical shift region of 3.5 ppm in Figure 8A. The intensity of these peaks decreases with time as can be seen in Figure 8C.

One can also observe the appearance of a cross peak at 1.7 and 3.1 ppm which corresponds to correlation of H^α^ and H^β^ of putrescine.

The z-TOCSY TR-NUS experiment was repeated several times, each time varying the concentration of face tonic and keeping the density of the bacteria and the concentration of glucose constant (as described in Section 2.4). It was observed that in each case the intensity of the glucose peaks was decreasing and the intensity of putrescine cross-peak was increasing, but the rate of change was different. The cross peaks corresponding to glucose at coordinates (3.46–4.6, 3.39–4.6, 3.23–4.6 ppm) in the z-TOCSY spectra were integrated and averaged in each case to describe the difference in kinetics.

**Figure 9:**
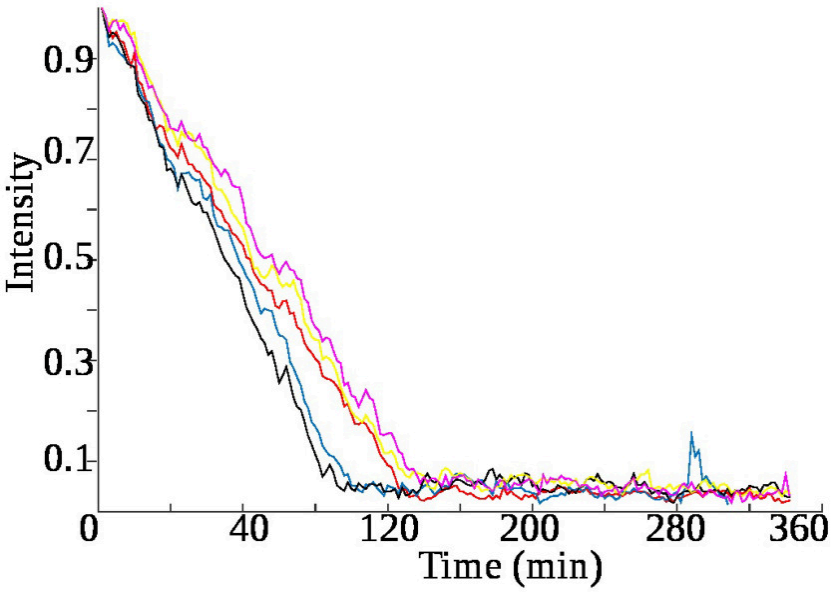
Rate of consumption of glucose varies with the amount of face tonic present in the sample. Each time glucose is 0.01% while face tonic is varied as : red–0%, black–0.1%, blue–0.2%, yellow–0.3%, pink–0.4%. In each case the trend line is obtained by taking the average of the peak integration values of three glucose cross peaks present at coordinates : (3.46–4.6, 3.39–4.6, 3.23–4.6 ppm) in the z-TOCSY spectra.

Figure 9 shows that in low concentrations (0.1, 0.2%) of the face tonic, the rate of consumption of glucose is much faster, than in the absence or in the case of high concentrations (0.3, 0.4%) of face tonic. This implies that at low concentrations the face tonic aids bacterial growth, while the acid stress caused by the face tonic takes effect only at high concentrations.

**Figure 10:**
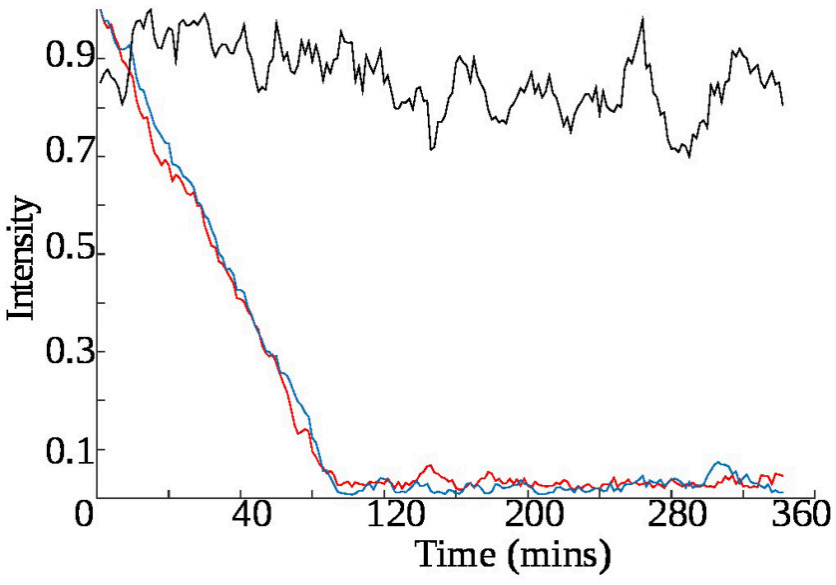
Rate of consumption of glucose varies with the amount of mandelic acid present in the sample. In each case the trend line is obtained by taking the normalized average of the peak integration values of three glucose cross peaks in the z-TOCSY spectra present at coordinates : 3.4–4.6, 3.39–4.6, 3.23–4.6 ppm are integrated and averaged. In the sample each time glucose is 0.01% while mandelic acid is varied as : red–0%, blue–0.002%, black–0.004%.

Similar experiments were conducted with other ingredients of the face tonic to compare their effect on the glucose metabolism by bacteria. The first series of experiments were carried out at growing concentrations of mandelic acid, which is a key ingredient of the face tonic (2%). Figure 10 shows the rate of consumption of glucose after addition of varying amounts of mandelic acid. The results show that the rate of consumption of glucose decreases with increasing concentration of mandelic acid. From the plots it can be easily concluded that 0.004% of mandelic acid (0.263 mM) was enough to inhibit cell metabolism as no glucose consumption takes place in this case.

Effect of other chemicals generally used in pharmaceuticals–lappa root extract, extract from willow bark and lanicera extract, have been studied by time-resolved NMR in a similar manner. The data from these experiments are presented in the Supplementary Material.

**Figure 11:**
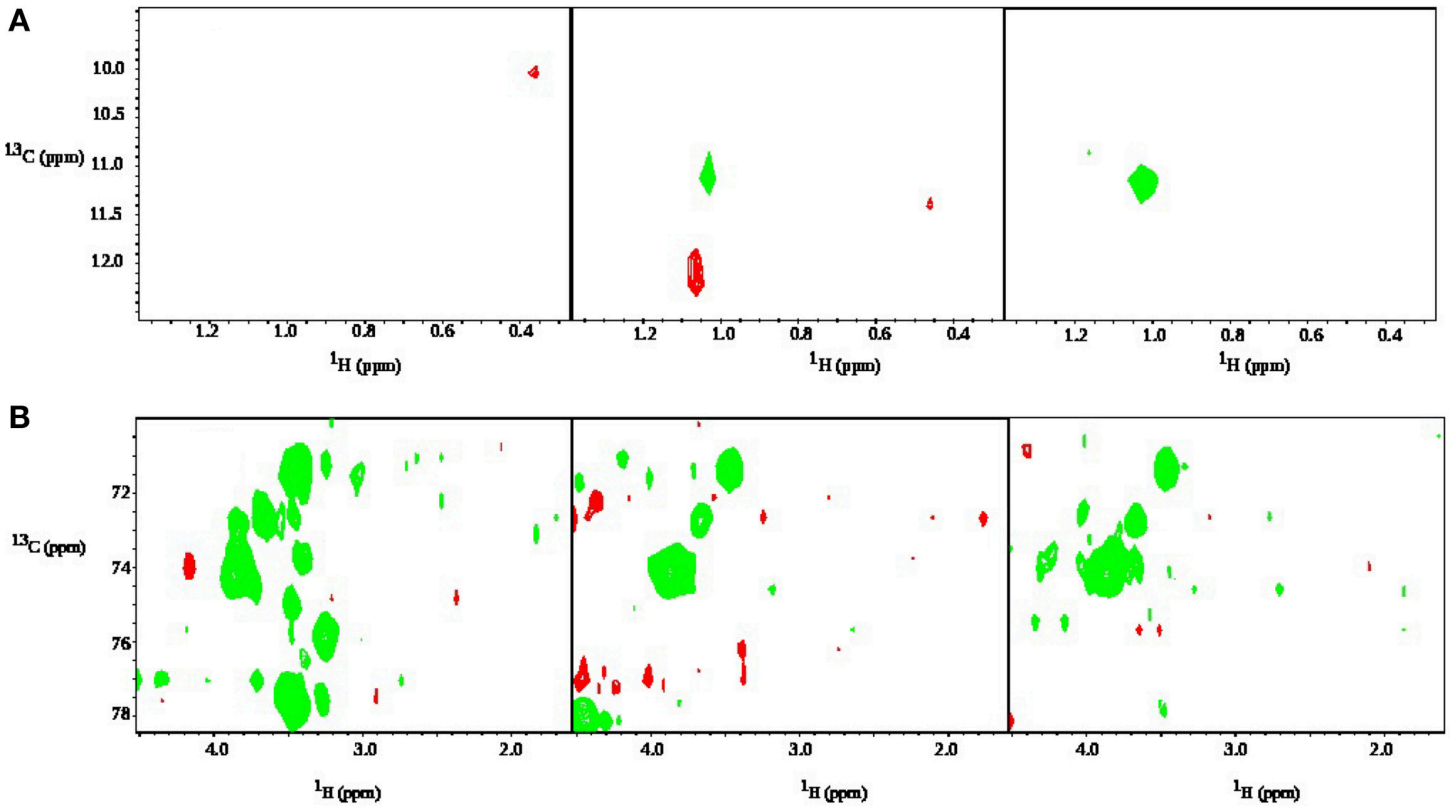
Zoomed-in regions of time-resolved HSQC spectra of a sample of bacteria with 0.01% of glucose and 0.1% of face tonic. Panels correspond to (from the left): 9 min, 9.5 h, 19 h after addition of both agents. In **(A)** one can see the product of glucose metabolism: peak from a methyl group of propionic acid growing with time. In **(B)** one can see that glucose and trehalose peaks are present. The peaks from glucose decrease in intensity with time.

**Figure 12:**
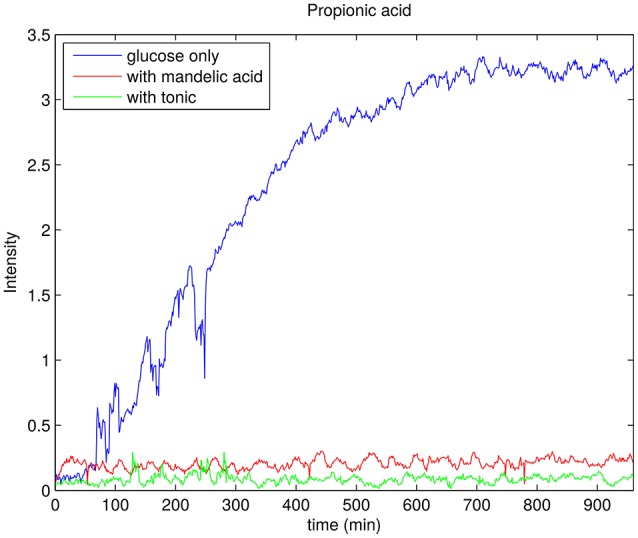
Rate of production of propionic acid for *P. acnes* sample with glucose only (blue), 0.002% of mandelic acid (red), and 0.1% of tonic (green). In each case the trend line is obtained by taking the peak integral values of propionic acid peak in ^13^C-HSQC spectrum.

To selectively monitor the products of glucose metabolism, time resolved HSQC experiments were implemented by using ^13^C glucose during the sample preparation. HSQC spectra of this sample is shown at three different time points in Figure 11. One can observe peaks from trehalose and the decrease in intensity of glucose peaks at chemical shift range 3–4 ppm in ^1^H dimension and 70–80 ppm in ^13^C dimension. A propionic acid peak is also seen to grow at 1 ppm (^1^H) and 11 ppm (^13^C) and is a direct product of glucose fermentation (Grinstead and Barefoot, 1992). Figure 12 shows time plot of the intensity of propionic acid peak in the sample without any anti-bacterial agents, with mandelic acid and with tonic.

## 4. Discussion

The presented method allows a precise and continuous monitoring of time-dependent processes in a biological sample. The effect of various anti-bacterial agents on bacteria could be monitored with a series of NMR experiments in time. However, for accurate detection of the changes occurring during the process one needs to resolve the various overlapping peaks. TOCSY was chosen as our primary experiment because of its ability to correlate all the protons in a spin system making structure elucidation possible. This experiment is generally sensitive because of the high natural abundance of magnetically active ^1^H isotope. We used the experiment in its optimized variant, providing cleaner spectra, known as z-TOCSY (Braunschweiler and Ernst, 1983). z-TOCSY is often used in identifying metabolites in mixtures of biological origin (see e.g., Massou et al., 2007).

The proposed extension of biological toolbox by 2D NMR experiments based on TR-NUS is not limited to z-TOCSY. To further enhance the selectivity of the technique one can use isotope labeling of the compounds to observe only particular metabolic pathways (Metallo et al., 2009; Chokkathukalam et al., 2014). Isotopic labeling allows also to use heteronuclear 2D experiments, i.e., those correlating hydrogen nuclei with e.g., magnetic isotopes of carbon (^13^C has spin $\frac{1}{2}$ and associated magnetic moment). The example of such a technique is Heteronuclear Single Quantum Correlation (HSQC) experiment (Bodenhausen and Ruben, 1980), used here to transfer coherence from ^1^H to ^13^C nuclei and back. Through our work we have shown the application of both z-TOCSY and ^13^C HSQC as good complementary experiments for process monitoring.

The *E. coli* spectra match with the results of study by Griengl (Griengl et al., 1999). Also, they show how ampicillin alters the metabolism. As can be seen from Figure 5 the products of cell decay, i.e., cadaverine and putrescine are produced in larger amounts when ampicillin is added. As it was recently shown, variety of bacterial antibiotics including ampicillin induce oxidative stress in *E. coli* cells (Belenky et al., 2015), and it is possible that polyamines, such as cadaverine and putrescine, known to protect *E. coli* from hazardous oxygen species were produced in these cells (Chattopadhyay et al., 2003). Interestingly, as seen from Figure 6, ampicillin alters the glucose metabolism toward more extensive lactic acid production at the slight expense of alcoholic fermentation. According to data collected by Belenky et al. (2015), treatment of bacteria with ampicillin for 90 min caused more than three-fold increase of acetyl-CoA abundance in cells. They also observed that concentration of intermediate metabolites of TCA cycle (e.g., citric acid, succinate, fumarate) increased in response to the presence of ampicillin. This observation is in line with our evidences, and may indicate that the larger number of NADH molecules produced by intense reduction of NAD^+^ by oxidative decarboxylation of pyruvate and TCA cycle is additionally regenerated (oxidized) thanks to lactic fermentation. The z-TOCSY and ^13^C HSQC serve as independent tests of repeatability. Comparing Figures 5, 6 shows, that the increased lactate and decreased ethanol production under the effect of ampicilin is observed in both tests, although with certain deviations. One has to note, however, that conditions of NMR measurements (e.g., temperature) can be controlled with high precision and differences are caused by other factors.

For *P. acnes*, we collected interesting data regarding glucose consumption in the presence of various concentrations of face tonic (Figure 9). While low concentration of face tonic (0.1–0.2%) slightly accelerated glucose consumption, the higher concentration of this agent restored utilization of glucose to the same level as in control. We hypothesize that face tonic which contains plant extracts added to bacterial suspension in low concentration supplements some nutritional substances and by this way enables a bit more intense glucose consumption. Figure 12 shows the rate of propionic acid production in *P. acnes*. Interestingly, 0.1% of tonic, which according to data presented in Figure 9 forced bacteria to somewhat faster glucose consumption, almost completely hindered the production of propionic acid. This may indicate that in the presence of mandelic acid (0.002%) or face tonic (0.1%) *P. acnes* cells use alternative pathway for ATP synthesis which does not result in production of propionic acid. Brzuszkiewicz et al. (2011) reported that even under anaerobic conditions all protein members of respiratory chain are present. For yet unknown reason, it is possible that fumarate respiration occurs in the presence of mandelic acid or components of face tonic. This process enables ATP production by F_0_F_1_ ATP synthase and can be accomplished by the presence of NADH dehydrogenase/complex I (NDH-1) and succinate dehydrogenase/fumarate reductase (SdhABC) whose expression in varying growth conditions was confirmed by Brzuszkiewicz et al. (2011). This pathway would explain our observations regarding glucose consumption without production of propionic acid in *P. acnes* samples. However, due to insufficient sensitivity, our measurements were not able to confirm the presence of succinate which is a final product of fumarate respiration in *P. acnes*.

We have to emphasize, that the presented method, although very selective, is able to monitor only main metabolites. Due to inherently low sensitivity of multidimensional NMR spectroscopy, less abundant products of metabolism can be found only in long measurements of the stable samples (Rolin et al., 1995; Deborde et al., 1998; Boyaval et al., 1999; Ye et al., 1999). Nevertheless, the uniqueness of molecular “fingerprints” in 2D spectra can make the method useful in many cases.

## 5. Conclusions

Nuclear magnetic resonance spectroscopy is a powerful tool of chemical analysis. It can be also applied in microbiology to monitor bacterial metabolism. However, studying bacterial samples that vary in time has been done so far only with simplest one-dimensional NMR techniques—since only they were fast enough to serve as snapshots of a process. Conventional two- and multi-dimensional experiments, although potentially can provide more unique fingerprints of molecules, are too slow for this purpose. We showed, that application of time-resolved non-uniform sampling makes it possible to use two-dimensional experiments of high resolution in a time-resolved manner. We believe, that our method may find its applications in studies of bacterial activity in a presence of complex mixtures.

## Author contributions

RD performed NMR experiments, analyzed results, and wrote a major part of manuscript. KG and TI prepared bacterial samples and reviewed the manuscript. RD has chosen and provided the antibacterial agents and reviewed the manuscript. MN performed *E. coli* experiments. KK coordinated the study, wrote part of the manuscript, and analyzed part of the NMR results.

### Conflict of interest statement

The authors declare that the research was conducted in the absence of any commercial or financial relationships that could be construed as a potential conflict of interest.
