# Supplementary material for: Fast 2D NMR Spectroscopy for In vivo Monitoring of Bacterial Metabolism in Complex Mixtures
Source: Front Microbiol. 2017 Jul 14;8:1306. doi: 10.3389/fmicb.2017.01306 (PMC5509914; doi:10.3389/fmicb.2017.01306)
Supplement: Supplementary file 6 [file DataSheet1.PDF]

Supplementary Material: Fast two-dimensional NMR spectroscopy for *in vivo* monitoring of bacterial metabolism in complex mixtures

Rupashree Dass, Katarzyna Grudziąż, Takao Ishikawa, Michał Nowakowski, Renata Dębowska , and Krzysztof Kazimierczuk

*Correspondence:
Author Name: Krzysztof Kazimierczuk
k.kazimierczuk@cent.uw.edu.pl

1 SUPPLEMENTARY DATA

1.1 Video of TR-NUS zTOCSY

The video file showing the spectral changes caused in a bacterial sample containing 0.01% glucose solution and 0.2% face tonic is available online.

1.2 Glucose metabolism in the presence of agents typically present in pharmaceutical face products

TR-NUS experiments implemented to z-TOCSY were conducted on bacterial samples containing laniceric acid, lappa root extract and willow bark extract. Sample preparation and data processing steps have been discussed in main text. It is observed that glucose peak intensities decrease in time depending on the kind of agent present. In fig. S1 we show trendlines of glucose peak intensities for each case.

2 SUPPLEMENTARY TABLES AND FIGURES

2.1 Figures

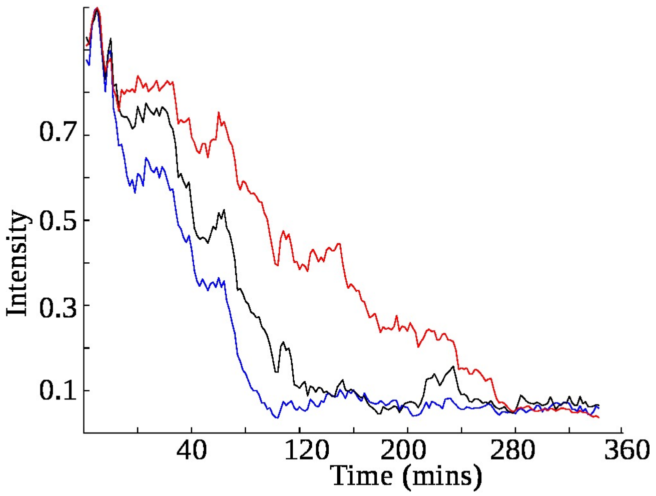

Figure S1: Rate of consumption of glucose varies with the agent present in the sample. Each time 0.6 μl of glucose was added. First sample contained 0.6 μl of laniceric acid (blue), second sample 0.6 μl of lappa root extract (black) and third sample 0.6 μl of willow bark extract (red). In each case the trend line is obtained by taking the average of the peak integration values of three glucose cross peaks in z-TOCSY spectrum present at coordinates : (3.46ppm - 4.6ppm, 3.39-4.6ppm, 3.23-4.6ppm)
